# Prognostic value of systemic inflammatory markers in ovarian Cancer: a PRISMA-compliant meta-analysis and systematic review

**DOI:** 10.1186/s12885-018-4318-5

**Published:** 2018-04-18

**Authors:** Ying Zhu, Sanqin Zhou, Yang Liu, Lingyun Zhai, Xiwen Sun

**Affiliations:** 1 Department of Gynecology, Second Affiliated Hospital, Zhejiang University School of Medicine, Hangzhou, 310009 China; 2 Department of Gynecology, Changxing people’s hospital, Huzhou, 313100 China; 3 Department of Obstetrics, Second Affiliated Hospital, Zhejiang University School of Medicine, Hangzhou, 310009 China; 4 Department of Gynecology and Obstetrics, Second Affiliated Hospital, Zhejiang University School of Medicine, Hangzhou, 310009 China

**Keywords:** Ovarian cancer, Inflammatory markers, Neutrophil-lymphocyte ratio, Platelet-lymphocyte ratio, Prognosis

## Abstract

**Background:**

The prognostic effect of elevated systemic inflammatory markers, including neutrophil-lymphocyte ratio (NLR) and platelet-lymphocyte ratio (PLR), remains controversial in cancer patients. This meta-analysis was conducted to evaluate the predictive values of these markers for prognoses in ovarian cancer patients.

**Methods:**

Potentially relevant publications in PubMed, ISI Web of Science, and EBSCO were searched. Pooled hazard ratios (HRs) and corresponding 95% confidence intervals (95% CIs) for overall survival (OS) and progression-free survival (PFS) were determined using a fixed or random effects model.

**Results:**

Ten studies involving 2919 patients were included in this meta-analysis. In multivariate analysis, the group with higher NLR had worse OS (HR = 1.34, 95% CI = 1.16-1.54) and shorter PFS (HR = 1.36, 95% CI = 1.17-1.57) than the control group. Furthermore, PLR values higher than the cut-off were associated with not only poorer OS (HR = 1.97, 95% CI = 1.61-2.40) but also more unfavorable PFS (HR = 1.79, 95% CI = 1.46-2.20). Univariate analysis also indicated the same results. Additionally, subgroup analysis showed that when the cut-off values for NLR and PLR were higher, their predictive effects became stronger.

**Conclusion:**

This comprehensive meta-analysis suggested that the values of inflammatory markers such as NLR and PLR were associated with ovarian cancer survival. Therefore, inflammatory markers can potentially serve as prognostic biomarkers.

**Electronic supplementary material:**

The online version of this article (10.1186/s12885-018-4318-5) contains supplementary material, which is available to authorized users.

## Background

Ovarian cancer is a commonly diagnosed gynecologic malignancy and the leading cause of gynecological cancer death in women [1]. Due to the lack of effective screening measures for the early detection of ovarian cancer, nearly 70% of ovarian cancer patients are diagnosed at an advanced stage. The prognosis of ovarian cancer remains poor; the 5-year survival rate is approximately 45.6% overall and approximately 25% for stage III and IV disease [2]. Therefore, the identification of many precise and feasible prognostic factors to guide personalized treatment and predict long-term survival of ovarian cancer patients is urgently needed. Traditional studies focus on the oncological characteristics of the tumor itself (such as tumor histology, grade and gene mutations), but recently, studies have begun to investigate host responses and the tumor microenvironment (such as immune cells, fibrocytes and metabolites) [3]. Systemic inflammatory responses are closely associated with cancer initiation, progression and metastasis, and thus, inflammatory markers, including the neutrophil-lymphocyte ratio (NLR) and platelet-lymphocyte ratio (PLR), have been studied and found to be related to cancer mortality and employed as useful prognostic indicators in many solid tumors [4–6]. A meta-analysis involving 1540 esophageal cancer patients demonstrated that elevated NLR (HR = 1.40, 95% CI = 1.08–1.81) and PLR (HR = 1.59, 95% CI = 1.14–2.21) were markedly associated with worse overall survival (OS) and that high NLR also predicted poor OS (HR = 1.63, 95% CI = 1.41–1.88) for prostate cancer [7, 8]. Additionally, high NLR values before treatment predicted unfavorable OS (HR = 1.81, 95% CI = 1.50–2.19) and PFS in colorectal cancer (HR = 2.10, 95% CI = 1.55–2.84) [9]. Moreover, Miao and colleagues assessed the predictive value of NLR and PLR in ovarian cancer patients, and the results suggested that patients with lower values of NLR (NLR < 3.02) or PLR (PLR < 207) had longer PFS and OS, and thus, NLR and PLR are significantly related to long-term survival in ovarian cancer [10]. However, a handful of studies demonstrated NLR and PLR as ineffective markers for predicting ovarian cancer prognosis. Raungkaewmanee et al. conducted a retrospective study on 166 epithelial ovarian cancer patients and found that NLR and PLR were not significantly associated with OS or PFS [11]. These contradictory studies thus cannot confirm the predictive effect of inflammatory markers on ovarian cancer survival. Hence, we conducted a systematic review and meta-analysis of published research data to evaluate the predictive value of NLR and PLR in the prognosis for ovarian cancer patients.

## Methods

The present meta-analysis was performed to assess the relationship between pretreatment NLR and PLR and ovarian cancer prognosis according to the protocol of Preferred Reporting Items for Systematic Reviews and Meta-Analysis (PRISMA) [12]. Furthermore, because this study is a meta-analysis without directly involving the handing or inclusion of personal data, ethical approval was not necessary.

### Search strategy

A comprehensive literature search for potentially relevant publications was performed by systematically searching the ISI Web of Science, PubMed and EBSCO using the search terms “neutrophil lymphocyte ratio” (OR “platelet lymphocyte ratio”) AND “ovarian cancer” (OR “ovarian carcinoma”) AND “prognosis” (OR “overall survival” OR “progression-free survival”). All databases were searched from January 2000 to April 2017, and the search was updated on November 2017. Bibliographies in each candidate publication were also searched to identify other potentially eligible studies.

### Selection criteria

Two independent investigators carefully screened the candidate publications. Studies were considered eligible if they satisfied all of the following criteria: (i) original articles including patients with histopathologically diagnosed ovarian cancer; (ii) studies providing pretreatment values of relevant inflammatory markers (NLR and PLR) and cut-off values; and (iii) studies reporting the relationship between pretreatment NLR and PLR and prognostic outcomes, with enough data to analyze hazard ratio (HR) and 95% confidence interval (CI) for PFS or OS. Overlapping or duplicate articles, review articles, letters, case reports, conference abstracts and laboratory studies based on animal models or cancer cell lines were eliminated.

### Data extraction and quality assessment

Information from the included publications was carefully extracted by two independent authors. In addition, any conflict was addressed by joint consensus. The following correlative information was collected in a predefined table from the eligible articles: general information (first author, publication year, and study country), patient characteristics (age, sample size, follow-up duration, cut-off value, and treatment methods) and endpoint parameters (OS and PFS). Many publications showed survival data using the Kaplan–Meier curves, and thus, GetData Graph Digitizer 2.25 (http://getdata-graph-digitizer.com/) was used to extract the relevant digitized survival data. Two investigators used the Newcastle-Ottawa-Scale (NOS) to evaluate the quality of the included study, and articles with NOS scores≥6 were defined as high-quality studies.

### Statistical analysis

Survival outcomes, including OS and PFS, were the primary end-points in this study. Thus, the logarithmic values of HR with 95% CI were calculated as the primary summary statistic. Heterogeneity among studies was determined using the Cochran Q-test and I-squared test. I^2^ > 50% and *p* < 0.10 were considered indicators of obvious heterogeneity, and a random effects model was used; otherwise, a fixed effects model was employed. The sources of heterogeneity in the included studies were determined using subgroup analysis and sensitivity analysis. Additionally, funnel plots with Egger’s and Begg’s tests were adopted for the precise assessment of potential publication bias.

STATA 12.0 software (STATA Corporation, College Station, TX, http://www.stata.com) was employed for all of the statistical analyses, and *p*-values< 0.05 were considered statistically significant.

## Results

### Study characteristics

**Fig. 1:**
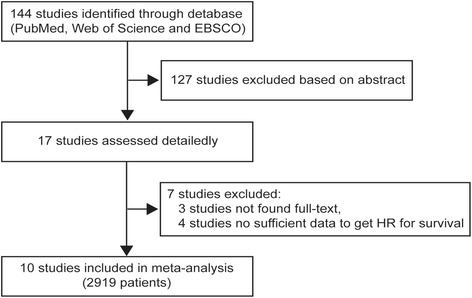
Flow diagram of study identification

A flow diagram representing the study identification procedure is shown in Fig. 1. The initial electronic search strategy yielded 144 potential studies after all duplicated data were removed. Among these, 127 articles were excluded after the screening of titles and abstracts. For further assessment, 17 studies were excluded after full-texts were screened. Seven of these studies were excluded. Ultimately, ten retrospective observational cohort studies involving 2919 patients were selected for subsequent meta-analysis [10, 11, 13–20]. The detailed characteristics and quality assessment results of the eligible publications are shown in Table 1. Cut-off values used in the eligible studies for NLR and PLR varied from 187.6 to 300 and 2.6 to 4, respectively. Additionally, after the evaluation of quality of the included studies, NOS scores of all of the selected articles were > 6, suggesting high quality.

**Table 1:** Main characteristics of all the studies included in the meta-analysis

Study, (Author/year/Country)	Cancer traits	Treatment	Patients	Age,	Follow-up, months (Range)	Therapy Response/Survival Data		NOS Score
Miao/2016/China [10]	EOC; Serous papillary histology (*n* = 216, 62.8%); FIGO stage III (*n* = 168, 48.8%); platinum-resistant group (*n* = 96, 27.9%);	Comprehensive surgical staging or tumor debulking followed by 6 cycles of adjuvant chemotherapy.	344	55 (45–84)	72 (61-97)	PFS (HR) PLR (cut-off value: 207) Ua: 3.851 (2.958 to 5.014) Ma: 1.952 (1.430 to 2.662) NLR (cut-off value: 3.02) Ua: 5.093 (3.886 to 6.676) Ma: 1.733 (1.225 to 2.453)	OS (HR) PLR (cut-off value: 207) Ua: 3.863 (2.960 to 5.043) Ma: 2.167 (1.565 to 3.000) NLR (cut-off value: 3.02) Ua: 4.970 (3.794 to 6.511) Ma: 1.616 (1.138 to 2.297)	9
Wang/2015/China [12]	SOC; FIGO stage III-IV (*n* = 93, 73.8%)	Cytoreductive surgery including para-aortic and pelvic lymph node dissection followed by platinum-based chemotherapy.	126	NA	41.3 (3.3–70.4)	PFS (HR) PLR (cut-off value: 187.6) Ua: 1.306 (0.684–2.495) Ma: 1.127 (0.433–2.932) NLR (cut-off value: 2.65) Ua: 3.191 (1.514–6.728) Ma: 3.554 (1.389–9.096)	OS (HR) PLR (cut-off value: 187.6) Ua: 1.090 (0.510–2.329) Ma: 1.689 (0.590–4.835) NLR (cut-off value: 2.65) Ua: 3.775 (1.497–9.519) Ma: 5.302 (1.817–15.471	8
Cho/2009/Korea [13]	EOC; Serous papillary histology (*n* = 131, 68.2%); FIGO stage III-IV (*n* = 125, 65.1%)	Treatment according to NCCN guidelines.	192	51.8 ± 12.9	20.9	DFS *P* = 0.014	OS (HR) NLR (cut-off value: 2.6) Ua: 6.05 (1.77–20.71) Ma: 8.42 (1.09–64.84)	8
Wang/2016/China [14]	OC; EOC (*N* = 122 85.3%); FIGO stage III-IV (*n* = 89, 62.2%)	NA	143	52.3 ± 14.1		PFS (HR) PLR (cut-off value: 201) Ua: 1.43 (0.87–2.35) Ma: 1.71 (0.80—3.66) NLR (cut-off value: 3.43) Ua: 2.11 (1.29–3.46) Ma: 2.20 (1.03–4.70)	OS (HR) PLR (cut-off value: 201) Ua: 1.76 (1.02–3.06) Ma: 2.05 (0.85–4.95) NLR (cut-off value: 3.43) Ua: 2.90 (1.66–5.05) Ma: 3.37 (1.39–8.15)	8
Asher/2011/UK [15]	OC; Serous papillary histology (*n* = 117, 49.6%); FIGO stage III-IV (*n* = 141, 60%)	Surgery followed by chemotherapy	235	62 (24–90)	24.5 (0.3–191.1)	NA	OS (HR) PLR (cut-off value: 300) Ua: 1.863 (1.37–2.534) Ma: 1.698 (1.031–2.797) NLR (cut-off value: 4) Ua: 1.686 (1.231–2.310) Ma: 0.865 (0.521–1.437)	7
Williams/2014/USA [16]	OC; Serous papillary histology (*n* = 300, 58%); FIGO stage III-IV (*n* = 308, 61%)	NA	519	≥55 ys: 56%	68.4 (1-252)	NA	OS (HR) NLR (cut-off value: 3.6) Ua: 1.43 (1.13,1.81) Ma: 1.37 (1.06, 1.76)	7
Thavaramara/2011/Thailand [17]	EOC; FIGO stage III-IV (*n* = 61, 47.6%)	Gynecologic surgery followed by chemotherapy	129	49.6 ± 12.5	NA	PFS (HR) NLR (cut-off value: 2.8) Ua: NA Ma: 0.7 (0.3-1.4)	OS (HR) NLR (cut-off value: 2.8) Ua: NA Ma: 0.7 (0.3-1.6)	8
Raungkaewmanee/2014/ Thailand [11]	EOC; Serous papillary histology (*n* = 136, 81.9%); FIGO stage III-IV (*n* = 78, 47%)	Surgery followed by chemotherapy	166	53 (23-85)	28.3 (6.1-94.4)	PFS (HR) PLR (cut-off value: 200) Ua: 1.89 (1.24–2.88) Ma: 1.51 (0.88-2.61) PFS (HR) NLR (cut-off value: 2.6) Ua: 1.16 (0.91-1.49) Ma: NA	OS (HR) PLR (cut-off value: 200) Ua: 2.67 (1.43–4.98) Ma: 1.41 (0.77-2.56) OS (HR) NLR (cut-off value: 2.6) Ua: 1.19 (0.72-1.98) Ma: NA	9
Feng/2016/ China [18]	HGSC; FIGO stage III-IV (*n* = 800, 91.4%)	Primary staging or debulking surgery, and 66.9% patients followed by chemotherapy	875	56 (30–90)	29 (1–115)	PFS (HR) NLR (cut-off value: 3.24) Ua: NA Ma: 1.250 (1.052–1.484)	OS (HR) NLR (cut-off value: 3.24) Ua: NA Ma: 1.189 (0.940–1.504)	8
Zhang/2015/ China [19]	OC; FIGO stage III-IV (*n* = 137, 72.1%)	cytoreductive surgery followed by platinum-based chemotherapy	190	51 (24–76)	43 (2–164)	PFS (HR) PLR (cut-off value: 203) Ua: 2.224 (1.626–3.042) Ma: 1.852 (1.271–2.697) NLR (cut-off value: 3.4) Ua: 2.012 1.476–2.741 Ma: NA	OS (HR) PLR (cut-off value: 203) Ua: 2.490 (1.758–3.527) Ma: 2.158 (1.468–3.171) NLR (cut-off value: 3.4) Ua: 2.172 (1.545–3.054) Ma: NA	9

*PFS* progression-free survival, *OS* overall survival, *CI* confidence interval, *OC* ovarian cancer, *EOC* epithelial ovarian cancer, *HGSC* high-grade serous ovarian cancer, *HR* hazard ratio, *NLR* neutrophil to lymphocyte ratio, *PLR* platelet to lymphocyte ratio, *ROC* receiver operating characteristic, *AUC* area under curve, *SOC* serous ovarian cancer, *NCCN* National Comprehensive Cancer Network, *NA* none acquired

### Association between NLR and ovarian cancer survival

**Fig. 2:**
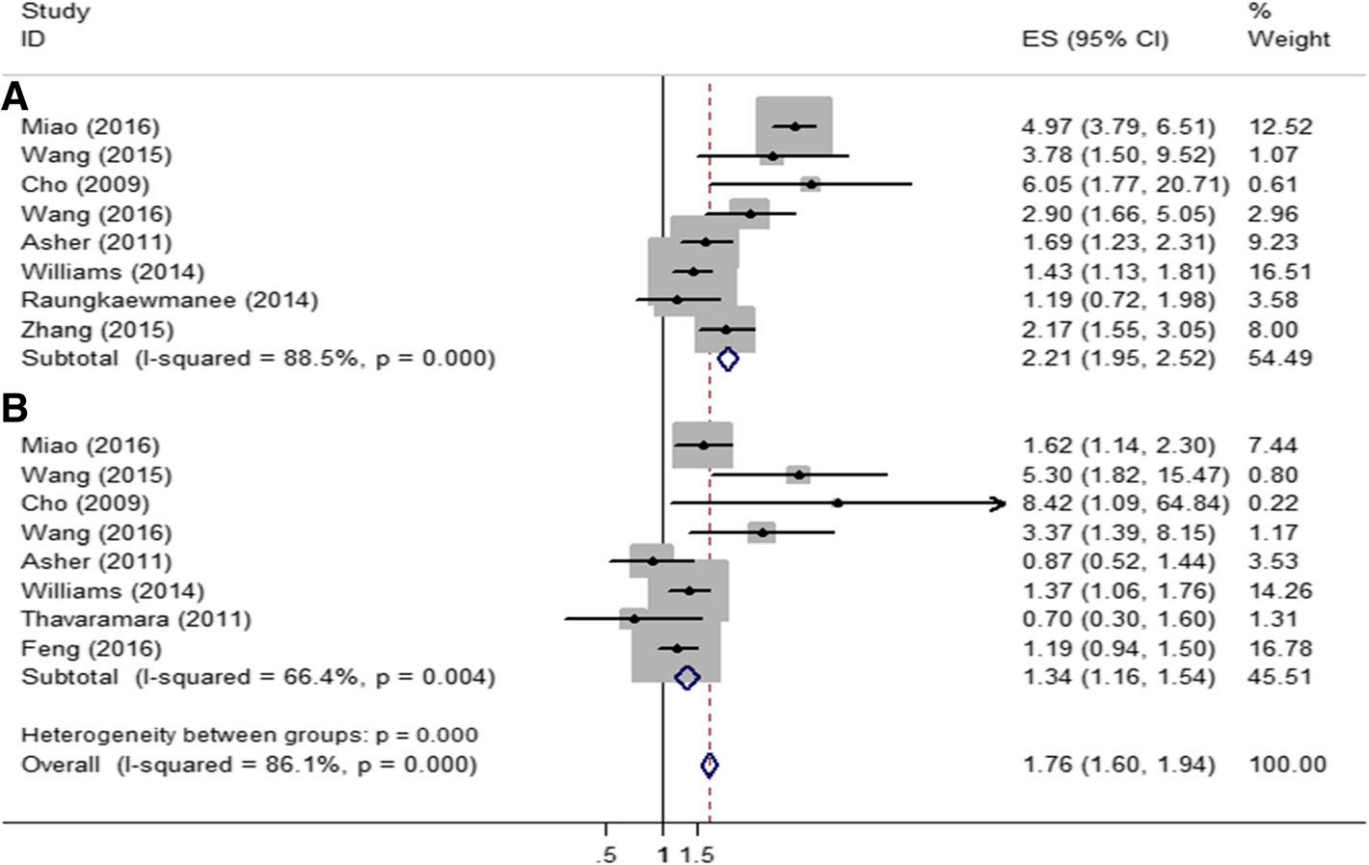
Forest plots showing the association between NLR and overall survival among ovarian cancer patients. **a** In multivariate model; (**b**) In univariate model

**Fig. 3:**
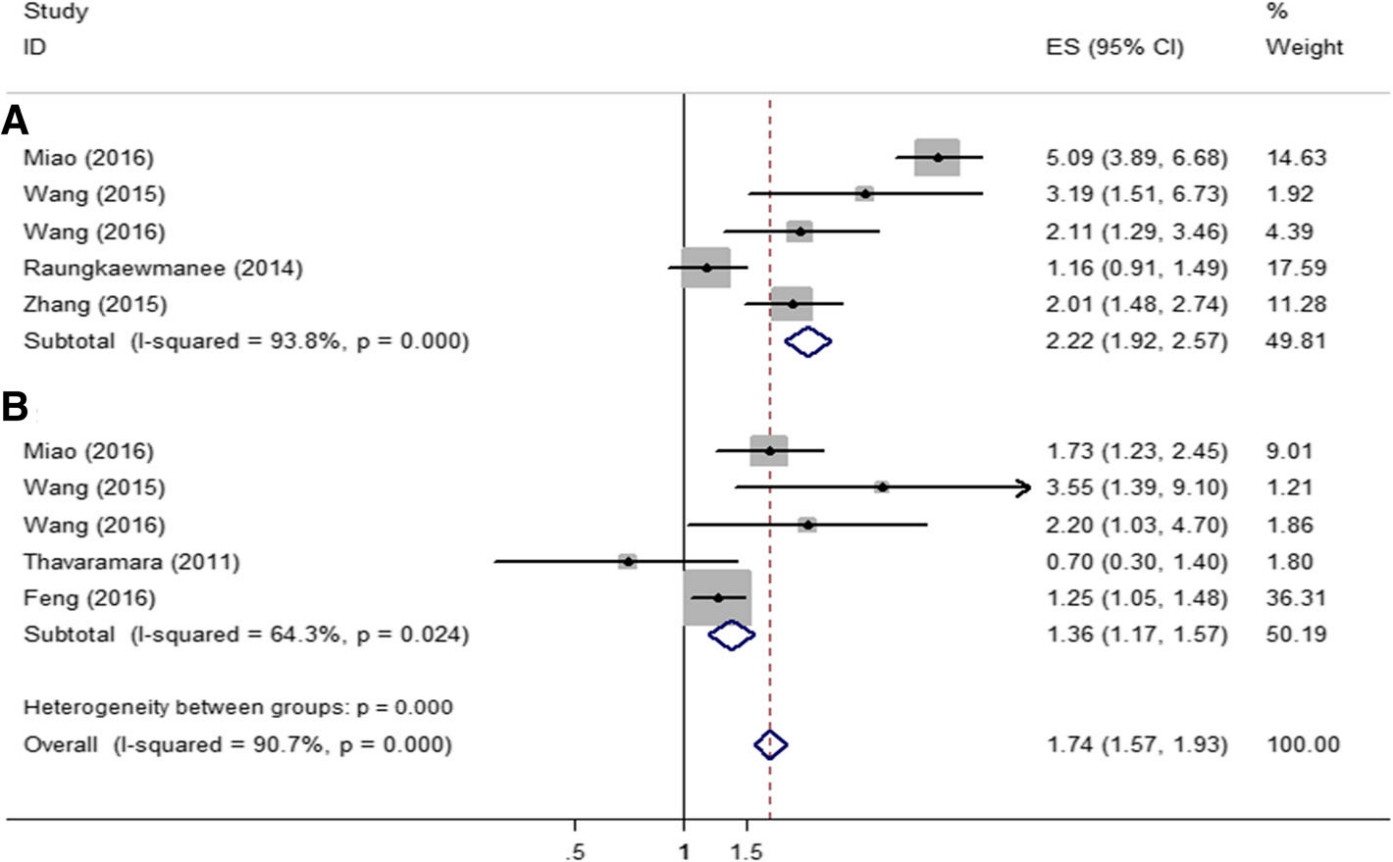
Forest plots showing the association between NLR and progression-free survival among ovarian cancer patients. **a** In multivariate model; (**b**) In univariate model

In univariate analysis, pooled estimates from five datasets revealed that elevated NLR significantly increased the risk of shorter OS (meta-hazard ratio [metaHR] = 2.21, 95% CI = 1.95-2.52) (Fig. 2a) and shorter PFS (metaHR = 2.22, 95% CI = 1.92-2.57) (Fig. 3a). For multivariate analysis, five studies were selected, and the metaHR of 1.34 (95% CI = 1.16-1.54) indicated that patients with higher NLR have worse OS (Fig. 2b). The synthesized estimates analysis also predicted that the group with elevated NLR had shorter PFS than the control group (metaHR = 1.36, 95% CI = 1.17-1.57) (Fig. 3b).

### Association between PLR and ovarian cancer survival

**Fig. 4:**
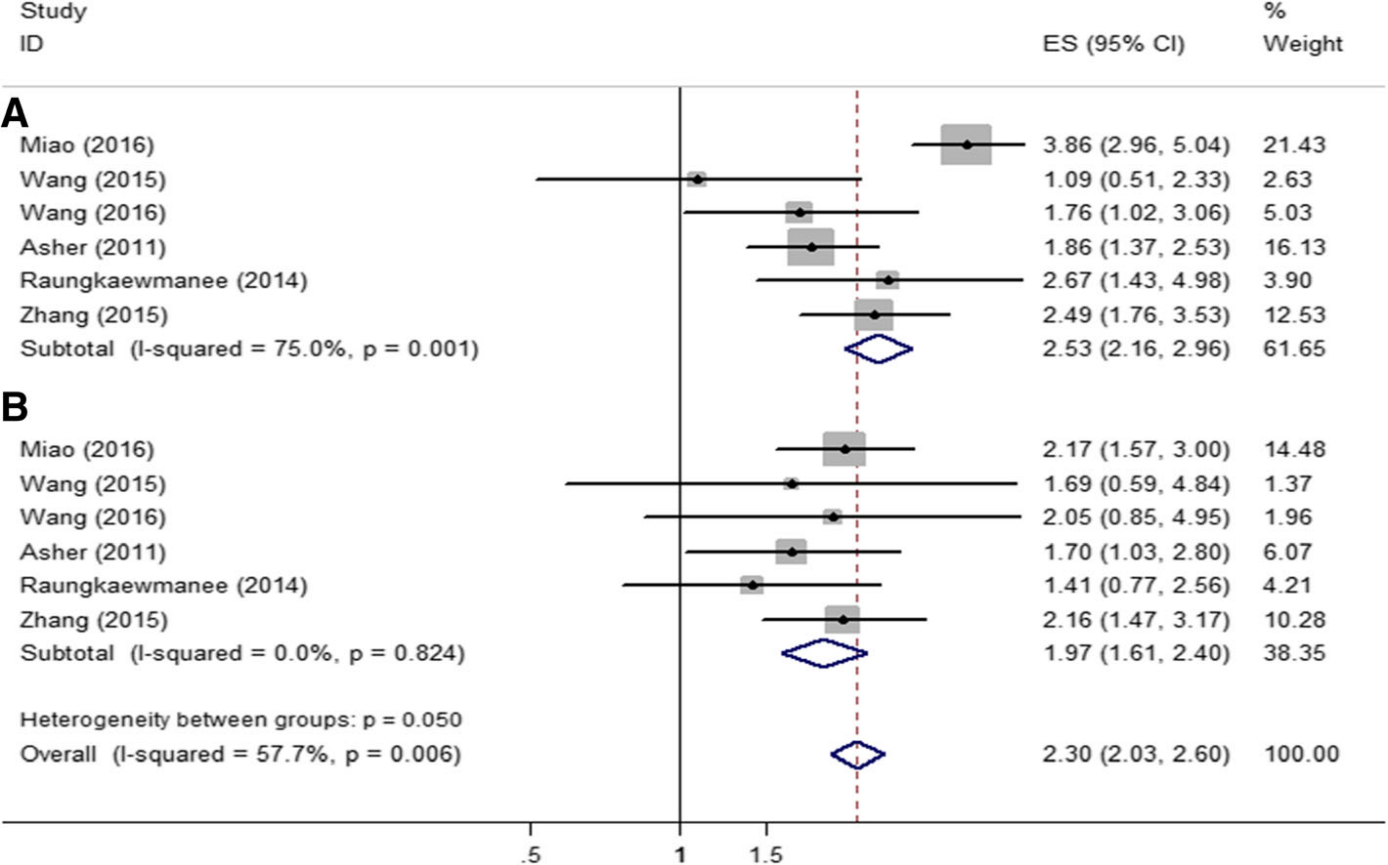
Meta-analysis of impact of PNR on ovarall survival of patients with ovarian cancer. **a** In multivariate model; (**b**) In univariate model

**Fig. 5:**
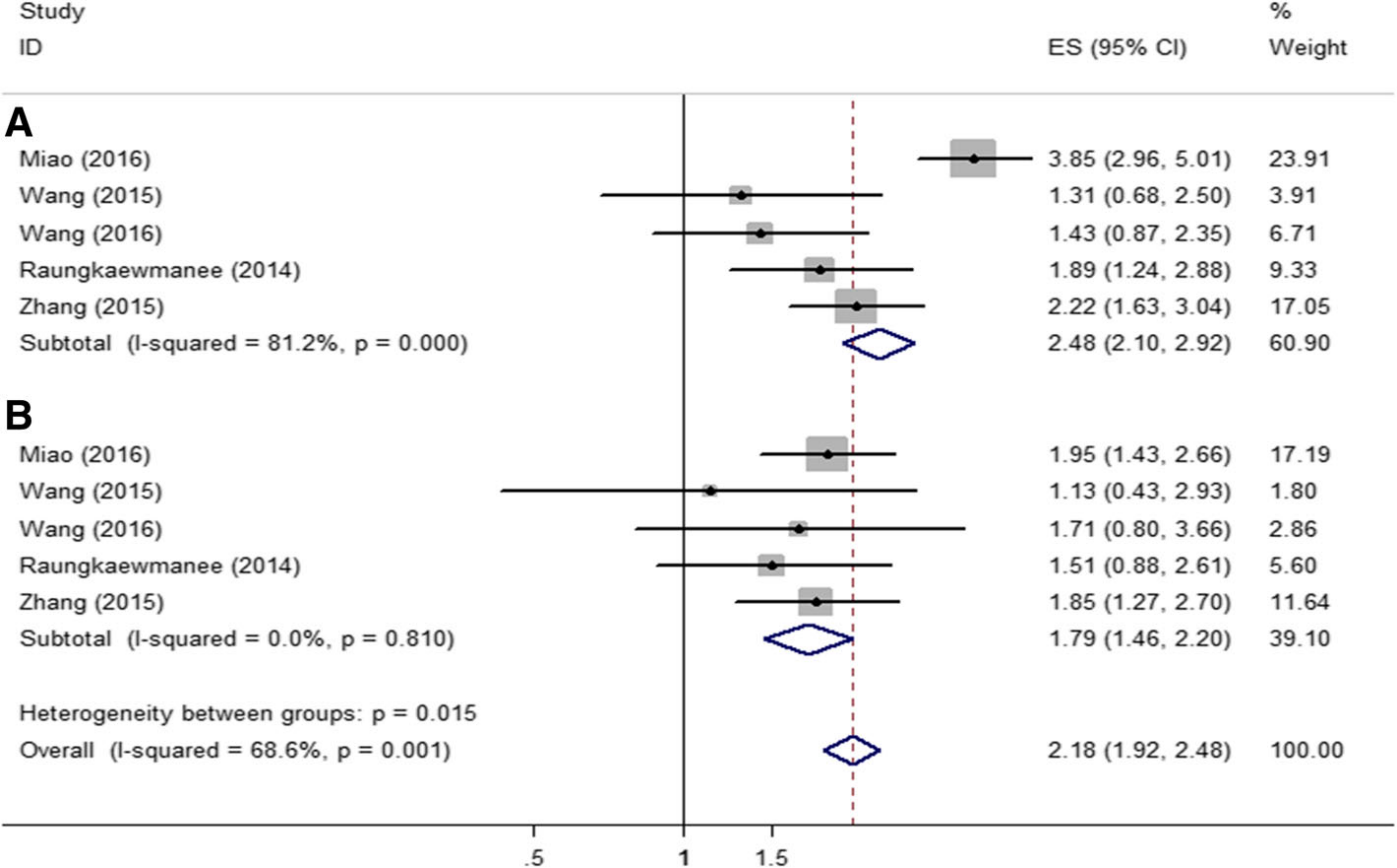
Meta-analysis of impact of PNR on progression-free survival of patients with ovarian cancer. **a** In multivariate model; (**b**) In univariate model

Six articles involving 1204 patients calculated hazard ratios of PLR for OS. Combined analysis showed that PLR values higher than the cut-off were associated with a worse OS (metaHR = 2.53, 95% CI = 2.16-2.96) in both univariate and multivariate analyses (metaHR = 1.97, 95% CI = 1.61-2.40) (Fig. 4a-b). Pooled analysis of 969 patients included in five studies showed that higher level of PLR indicated unfavorable PFS in both univariate (metaHR = 2.48, 95% CI = 2.10-2.96) and multivariate analyses (metaHR = 1.79, 95% CI = 1.46-2.20) (Fig. 5a-b).

### Subgroup analysis

In some analysis, there was significant heterogeneity in both pooled HR of OS and PFS, and thus, subgroup analyses were conducted to identify the source of heterogeneity. Subgroups were stratified based on cut-off values (NLR: > 3 vs. ≤3; PLR: > 200 vs. ≤200) and sample size (> 200 vs. ≤200) to evaluate HR of OS and PFS. In multivariate analysis, the heterogeneity was noticeable for low cut-off value and small sample size. However, similar results were not obtained using univariate analysis (Tables 2 and 3). Subgroup analysis was also performed according to the subtype of ovarian cancer, the prognostic value of NLR and PLR not only found in whole ovarian cancer, but also in epithelial ovarian cancer (Additional file 1: Table S1).

**Table 2:** Subgroup analysis results of NLR and ovarian cancer survival (OS and PFS)

Subgroup Analysis	Univariate analysis		Heterogeneity P1 value	Multivariate analysis		Heterogeneity P2 value
	NO.	ES (95% CI)		NO.	ES (95% CI)	
NLR for OS	8	2.21 (1.95-2.52)	0.000	8	1.34 (1.16-1.54)	0.004
Subgroup 1: cut-off						
> 200	5	2.26 (1.97-2.59)	0.000	5	1.32 (1.14-1.52)	0.060
≤ 200	3	1.82 (1.20-2.76)	0.012	3	1.78 (0.95-3.32)	0.004
Supgroup 2: sample size						
> 200	3	2.24 (1.92-2.61)	0.000	3	1.35 (1.12-1.63)	0.142
≤ 200	5	2.16 (1.70-2.74)	0.033	5	1.32 (1.07-1.64)	0.002
NLR for PFS	5	2.22 (1.92-2.57)	0.000		1.36 (1.17-1.57)	0.024
Subgroup 1: cut-off						
> 200	3	3.17 (2.63-3.83)	0.000		1.36 (1.17-1.58)	0.114
≤ 200	2	1.28 (1.01-1.62)	0.012		1.34 (0.74-2.44)	0.009
Supgroup 2: sample size						
> 200	1	5.09 (3.89-6.68)	/		1.33 (1.14-1.55)	0.098
≤ 200	4	1.58 (1.32-1.88)	0.004		1.62 (1.02-2.59)	0.020

**Table 3:** Subgroup analysis results of PLR and ovarian cancer survival (OS and PFS)

Subgroup Analysis	Univariate analysis		Heterogeneity P1 value	Multivariate analysis		Heterogeneity P2 value
	NO.	ES (95% CI)		NO.	ES (95% CI)	
PLR for OS	6	2.53 (2.16-2.96)	0.001	6	1.97 (1.61-2.40)	0.824
Subgroup 1: cut-off						
> 200	4	2.63 (2.23-3.10)	0.002	4	2.06 (1.16-2.56)	0.867
≤ 200	2	1.86 (1.15-3.01)	0.74	2	1.47 (0.87-2.48)	0.770
Supgroup 2: sample size						
> 200	2	2.82 (2.31-3.45)	0.000	2	2.02 (1.54-2.65)	0.422
≤ 200	4	2.14 (1.67-2.75)	0.193	4	1.91 (1.42-2.55)	0.694
PLR for PFS	5	2.48 (2.10-2.92)	0.000	5	1.79 (1.46-2.20)	0.810
Subgroup 1: cut-off						
> 200	3	2.75 (2.28-3.31)	0.001	3	1.89 (1.50-2.38)	0.943
≤ 200	2	1.70 (1.19-2.42)	0.354	2	1.41 (0.88-2.66)	0.606
Supgroup 2: sample size						
> 200	1	3.85 (2.96-5.01)	/	1	1.95 (1.43-2.66)	/
≤ 200	4	1.86 (1.51-2.30)	0.330	4	1.67 (1.27-2.20)	0.788

### Publication bias and sensitivity analysis

Visual inspection of the funnel plot was performed to determine publication bias in the included studies, and the results revealed evident symmetry for NLR and PLR regarding OS and PFS analyses, suggesting the lack of obvious publication bias(Additional file 2: Figure S2). These results were also confirmed using the Begg’s and Egger’s tests. Furthermore, a sensitivity analysis was performed by assessing the potential impact of each article on pooled HRs. The results showed that no studies had excessive influence on the stability of the pooled effect of comparisons. Therefore, this meta-analysis is robust.

## Discussion

Currently, ovarian cancer survival rates are very variable even among patients with similar disease stage and tumor subtype receiving same therapy. Therefore, prognostic biomarkers are urgently needed to assist the precise prediction of patient prognosis and to facilitate the identification novel therapeutic targets. Many publications have reported the predictive value of inflammatory markers (NLR and PLR) for prognosis in ovarian cancer patients, but the results have been contradictory. This present study is the first meta-analysis assessing the prognostic value of inflammatory markers (NLR and PLR) for long-term survival (OS and PFS) in ovarian cancer patients. Overall, the pooled data provided evidence that both higher NLR and higher PLR predicted inferior survival outcomes. The latest study conducted by Li’s group also indicted the predict role [21]. The subgroup analysis also showed that when the cut-off values for NLR and PLR were higher, their predictive effects became stronger.

Many hematological parameters such as neutrophil count, monocyte count, platelet count, NLR and PLR are systemic inflammatory parameters that reflect systemic inflammation and organ dysfunction. NLR, one of markers of systemic inflammation, may represent the pro-angiogenic/pro-inflammatory status in tumor tissues as well as the ratio between neutrophils and lymphocytes, thereby reflecting patients’ immune functions. Elevated NLR in patients might indirectly indicate poor lymphocyte-mediated immune response against tumors, leading to accelerated tumor progression and poor prognosis. Preclinical studies show that neutrophils may act through transforming growth factor β (TGF-β)-mediated signaling pathways, which can promote tumor cell proliferation [5]. PLR, another indicator with a very important effect on the pathogenesis of systemic inflammatory response, has also been shown to be associated with the prognosis of cancer patients. Platelet counts may be elevated because of the release of inflammatory mediators by tumors or inflammatory cells leading to the stimulation of megakaryocytes to produce platelets. Platelets are also considered to be the major sources of various cytokines, such as vascular endothelial growth factor, that are important for tumor angiogenesis. Clinical studies have indicated NLR and PLR as indicators with prognostic implications in many types of cancer, such as breast cancer [22], kidney cancer [23], pancreatic cancer [24], and lung cancer [6]. Studies have also reported that changes in NLR and PLR values are associated with pathological response to neoadjuvant chemotherapy or preoperative chemoradiotherapy in gastric, esophageal and colorectal cancer [25]. Therefore, systemic inflammatory markers have the potential to be used as valuable clinical parameters for predicting chemotherapeutic efficacy and prognosis. It is known that NLR and PLR values can be easily determined from blood tests, and thus, these parameters have great potential for clinical application in the diagnosis and therapy of solid tumors [26].

Nevertheless, this meta-analysis has several limitations. First, the eligible articles described retrospective observational cohort studies, and thus, the evidence was of low quality. Second, this pooled analysis included only ten studies involving 2919 patients, and the small sample size resulted in insufficient data preventing detailed subgroup analysis. Third, although NLR and PLR were easy to measure, the cut-off values were variable and may have been affected by some other conditions, such as bacterial infection and blood coagulation disorders. Therefore, some unexplainable heterogeneity may have existed among the studies.

## Conclusion

In summary, this comprehensive meta-analysis indicated that inflammatory markers such as NLR and PLR are associated with ovarian cancer survival. Therefore, inflammatory markers can potentially serve as prognostic biomarkers. However, to establish the clinical utility of inflammatory markers as prognostic indicators in ovarian cancer, large and well-designed prospective studies are required to confirm clinical significance and to define precise cut-off values for these markers.

## Additional files

Additional file 1: **Table S1.** Subgroup analysis results of NLR and PLR for ovarian cancer survival (OS and PFS). (DOCX 19 kb)

Additional file 2: **Figure S2.** Funnel plot analysis of potential publication bias. (A) NLR for overall survival; (B) NLR for progression-free survival; (C) PLR for overall survival; (D) PLR for progression-free survival. (TIFF 2573 kb)

## Abbreviations

95%CIs: 95% confidence intervals
HRs: Hazard ratios
NLR: Neutrophil-lymphocyte ratio
OS: Overall survival
PFS: Progression-free survival
PLR: Platelet-lymphocyte ratio
PRISMA: Preferred Reporting Items for Systematic Reviews and Meta-Analysis
