# Supplementary material for: Prognostic value of systemic inflammatory markers in ovarian Cancer: a PRISMA-compliant meta-analysis and systematic review
Source: BMC Cancer. 2018 Apr 18;18:443. doi: 10.1186/s12885-018-4318-5 (PMC5907305; doi:10.1186/s12885-018-4318-5)
Supplement: Supplementary file 1 — Table S1. Subgroup analysis results of NLR and PLR for ovarian cancer survival (OS and PFS). (DOCX 19 kb) [file 12885_2018_4318_MOESM1_ESM.docx]

**Table S1: Subgroup analysis results of NLR and PLR in ovarian cancer survival (OS and PFS)**

| Subgroup  Analysis | Univariate analysis | | Heterogeneity  P1 value | Multivariate analysis | | Heterogeneity  P2 value |
| --- | --- | --- | --- | --- | --- | --- |
|  | **NO.** | **ES (95% CI)** |  | **NO.** | **ES (95% CI)** |  |
| NLR for OS | 8 | 2.21 (1.95-2.52) | 0.000 | 8 | 1.34 (1.16-1.54) | 0.004 |
| Ovarian cancer | 4 | 1.73 (1.48-2.03) | 0.000 | 3 | 1.33 (1.07-1.66) | 0.031 |
| Epithelial ovarian cancer | 4 | 3.69 (2.94-4.63) | 0.000 | 5 | 1.34 (1.12-1.62) | 0.008 |
| NLR for PFS | 5 | 2.22 (1.92-2.57) | 0.000 | 5 | 1.36 (1.17-1.57) | 0.024 |
| Ovarian cancer | 1 | 2.11 (1.29-3.49) | - | 1 | 2.2 (1.17-1.57) | - |
| Epithelial ovarian cancer | 4 | 2.23 (1.92-2.61) | 0.000 | 4 | 1.33 (1.15-1.55) | 0.022 |
| PLR for OS | 6 | 2.53 (2.16-2.96) | 0.001 | 6 | 1.97 (1.61-2.40) | 0.824 |
| Ovarian cancer | 2 | 1.84 (1.4-2.4) | 0.002 | 2 | 1.78 (1.15-2.75) | 0.617 |
| Epithelial ovarian cancer | 4 | 3.00 (2.47-3.64) | 0.000 | 4 | 2.02 (1.6-2.75) | 0.717 |
| PLR for PFS | 5 | 2.48 (2.10-2.92) | 0.000 | 5 | 1.79 (1.46-2.20) | 0.810 |
| Ovarian cancer | 1 | 1.43 (0.87-2.35) | - | 1 | 1.71 (0.80-3.66) |  |
| Epithelial ovarian cancer | 4 | 2.65 (2.23-3.16) | 0.001 | 4 | 1.79 (1.45-2.22) | 0.664 |
