# Supplementary figures and images for: Prognostic value of systemic inflammatory markers in ovarian Cancer: a PRISMA-compliant meta-analysis and systematic review
Source: BMC Cancer. 2018 Apr 18;18:443. doi: 10.1186/s12885-018-4318-5 (PMC5907305; doi:10.1186/s12885-018-4318-5)

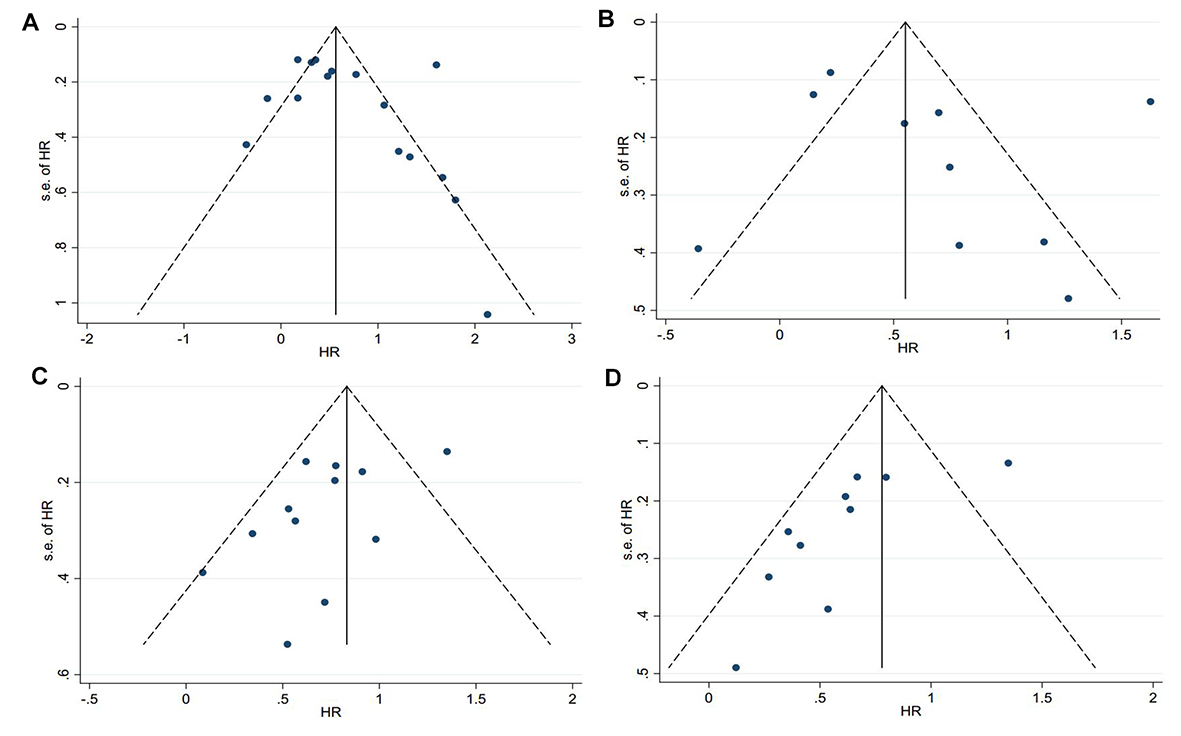

Supplement: Supplementary file 2 — Figure S2. Funnel plot analysis of potential publication bias. (A) NLR for overall survival; (B) NLR for progression-free survival; (C) PLR for overall survival; (D) PLR for progression-free survival. (TIFF 2573 kb) [file 12885_2018_4318_MOESM2_ESM.tif]
